# Neuronal Pnn Deficiency Increases Oxidative Stress and Exacerbates Cerebral Ischemia/Reperfusion Injury in Mice

**DOI:** 10.3390/antiox11030466

**Published:** 2022-02-26

**Authors:** Shu-Yuan Hsu, Chih-Hung Chen, Sujira Mukda, Steve Leu

**Affiliations:** 1 Department of Anatomy, Graduate Institute of Biomedical Sciences, College of Medicine, Chang Gung University, Taoyuan 33302, Taiwan; hsusy@mail.cgu.edu.tw; 2 Institute for Translational Research in Biomedicine, Kaohsiung Chang Gung Memorial Hospital, Kaohsiung 833401, Taiwan; 3 Department of Internal Medicine, Divisions of General Medicine, Kaohsiung Chang Gung Memorial Hospital and Chang Gung University College of Medicine, Kaohsiung 833401, Taiwan; totoro@adm.cgmh.org.tw; 4 Research Center for Neuroscience, Institute of Molecular Biosciences, Mahidol University, Salaya, Nakornpathom 73170, Thailand; sujira.muk@mahidol.ac.th; 5 Department of Biotechnology, College of Life Science, Kaohsiung Medical University, Kaohsiung 807378, Taiwan

**Keywords:** Pnn, neuron, ischemia/reperfusion, mRNA alternative splicing, oxidative stress

## Abstract

Cerebral stroke remains one of the leading causes of death worldwide. Ischemic stroke caused by the sudden loss of blood flow in brain is the major type of cerebral stroke. In addition to necrotic cell death in the ischemic core region, neuronal apoptosis is usually observed in the ischemic penumbra. Pnn, a multi-functional protein, participates in cellular proliferation, migration, differentiation, apoptosis as well as cell–cell interaction through its abilities in regulating gene transcription and mRNA processing. Our recent studies have demonstrated that Pnn has a cell type-specific distribution manner in neural cells under ischemic injury and plays a protective role in astrocytes against ischemic stress. In this study, we generated an inducible neuron-specific Pnn deficiency mouse model to further investigate the physiological role of Pnn in neurons. To directly examine the role of neuronal Pnn in ischemic stress, four weeks after induction of Pnn deficiency in neurons, middle cerebral artery occlusion (MCAO) was applied to induce cerebral ischemia/reperfusion in mice. In the cerebrum and hippocampus with neuronal Pnn depletion, the expression of SRSF2, a mRNA splicing regulator, was increased, while the expression of SRSF1, a functional antagonist of SRSF2, was reduced. Expression levels of ROS generators (NOX-1 and NOX-2) and antioxidant proteins (GR, HO-1, NQO-1) were upregulated in brain tissue with loss of neuronal Pnn, echoing an increase in oxidized proteins in cortical and hippocampal neurons. Furthermore, the expression of DNA damage marker, p53bp1, was found in the choroid plexus of mice with neuronal Pnn depletion. In mice with MCAO, compared to wild type mice, both increased cerebral infarcted area and elevated expressions of proapoptotic proteins were found in mice with neuronal Pnn depletion. In conclusion, Pnn deficiency increases oxidative stress in neurons and exacerbates cerebral ischemia/reperfusion injury in mice.

## 1. Introduction

Cerebral stroke is one of the leading causes of disability and mortality worldwide and is divided into ischemic or hemorrhagic stroke. Thrombotic or embolic cerebral infarction resulting from atherosclerotic obstruction or clot-induced embolisms in cervical and cerebral arteries are major types of ischemic stroke. Along with arterial occlusion, the core of brain tissue without blood flow supply is fatally injured and subsequently undergoes necrotic cell death [1,2]. Recent research has revealed that neurons in ischemic penumbra may undergo apoptosis several hours or days after injury, and thus they are potentially recoverable for some time after the onset of stroke [3,4]. Hence, neurons in the ischemic penumbra still have opportunity to survival after appropriate post-ischemic stroke therapy [1,2]. Therefore, the regulation of expression or activation of apoptosis-associated proteins is one of the potential strategies to prevent neuronal death after ischemic stroke.

Pnn, an SR (serine- and arginine- rich)-related protein, plays multiple roles in regulating cell proliferation, cell migration, cell–cell connection as well as cell differentiation through its capacities in modulating alternative splicing, transcriptional regulation, and protein–protein interaction [5,6,7,8,9,10,11,12]. Pnn has an RS (arginine/serine rich) domain (arginine-Serine) and no RNA recognition motif (RRM), indicating that Pnn may participate in RNA processing through its interaction with other RS domain-containing splicing regulators [12]. Our previous study with systemic Pnn deficiency showed that loss of Pnn results in early embryonic lethality [5], while in a mouse model with reduced Pnn expression, Pnn is found to be involved in the development of the small intestine, neural crest, dorsal dermis, and axial skeleton [8,13]. In a recent study with a zebrafish model, we also demonstrated that loss of Pnn expression inhibits proliferation and differentiation of neural crest cells [14]. Although the role of Pnn in embryonic development and regulating proliferation/differentiation/apoptosis in proliferative cells has been investigated in past years, the physiological role of Pnn in non-dividing and highly differentiated cells, particularly the post-mitotic neurons, remains unclear.

In our previous study, we indicated a high expression of Pnn in neurons and oligodendrocytes, but not in astrocytes [6]. Our recent studies also demonstrated that Pnn has a cell type-specific distribution manner in neural cells under ischemic injury and plays a protective role in astrocytes against ischemic stress [15]. Since Pnn is reported to be involved in apoptosis in breast carcinoma cells through regulating expression and alternative splicing in apoptosis-associated proteins, Pnn may also play a role in modulating neuronal apoptosis in cerebral ischemia. In this study, we used an animal model with gene manipulation and induction of ischemic stroke to reveal the role of Pnn in stress response to ischemic injury.

## 2. Materials and Methods

### 2.1. Ethics

All animal experimental procedures were approved by the Institute of Animal Care and Use Committee at Kaohsiung Chang Gung Memorial Hospital (no. 2015100501) and performed in accordance with the Guide for the Care and Use of Laboratory Animals (NIH publication no. 85-23, National Academy Press, Washington, DC, USA, revised 1996).

### 2.2. Generation of Neuron-Specific Pnn Depletion Mice

To generate the mouse model with inducible neuron-specific Pnn depletion, the mouse strain carrying a Pnn conditional allele (Pnn^flox/flox^) was crossed with the mouse strain carrying the Cre-ERT2 transgene driven by the CaMKII promoter (CaMKII-CreERT2, Cre recombinase specifically expressed in excitatory neurons and activated by tamoxifen). The genotyping was performed by PCR (polymerase chain reaction) on tail tip-extracted genomic DNA with primers targeted against the Pnn allele and CreERT2 expression cassette. To induce the loss of Pnn in adult neurons, 6-week-old CaMKII-CreERT2, Pnn^flox/flox^ mice were injected with tamoxifen (20 mg/kg/day) for 5 consecutive days.

### 2.3. Middle Cerebral Artery Occlusion

Four weeks after tamoxifen injection, mice were anesthetized with 2.0% inhalational isoflurane and supine on a warming pad (37 °C). After exposure of the left common carotid artery (LCCA) through a transverse neck incision, the vessel was permanently ligated. On the other hand, after exposure of the right common carotid artery (RCCA) through a transverse neck incision, a small incision was made in the RCCA. A nylon filament (0.028 mm in diameter) was carefully inserted into the distal right internal carotid artery through the RCCA incision for occlusion of the right middle cerebral artery (RMCA) to cause brain infarction. The nylon filament was removed 120 min after occlusion, followed by closure of the muscle and skin in layers.

### 2.4. Western Blot

Equal amounts (10–30 mg) of protein extracts from cerebral tissues or cells were loaded and separated by SDS-PAGE (sodium dodecyl sulfate polyacrylamide gel electrophoresis) using 8–12% acrylamide gradients. Following electrophoresis, separated proteins were transferred electrophoretically to a polyvinylidene difluoride (PVDF) membrane (Amersham Biosciences, Piscataway, NJ, USA). Non-specific proteins were blocked by incubating the membrane in blocking buffer (5% non-fat dry milk in T-TBS containing 0.05% Tween 20) overnight. The membranes were incubated with the indicated primary antibodies against Pnn (P3A, 1:1000, a kind gift from Prof. Pin Ouyang), SRSF1 (1:500, Santa Cruz, Santa Cruz, CA, USA), SRSF2 (1:500, Santa Cruz, Santa Cruz, CA, USA), GR (1:1000, Abcam, Cambridge, MA, USA), GPx (1:500, Abcam, Cambridge, MA, USA), HO-1 (1:1000, Sigma, St. Louis, MO, USA), NQO-1 (1:500, Abcam, Cambridge, MA, USA), NOX-1 (1:2000, St. Louis, MO, USA), NOX-2 (1:1000, St. Louis, MO, USA), p21 (1:500, Santa Cruz, CA, USA), TGF-β (1:500, Abcam, Cambridge, MA, USA), and actin (1:1000, Cell Signaling, Danvers, MA, USA) for 1 h at room temperature. Signals were detected with HRP-conjugated goat antimouse or goat ant-rabbit with ECL (Perkin Elmer, Waltham, MA, USA).

### 2.5. Histopathological and Immunofluorescent Staining

For immunofluorescent staining, isolated cerebral tissues were mounted in OCT (optimal cutting temperature compound) and used for preparing cryosections. Cryosections (10 μm) were fixed and permeated with acetone or 4% paraformaldehyde with 0.5% Triton X-100, and then incubated with antibodies against Pnn, SRSF1, SRSF2, and p53bp1 at 4 °C overnight. Sections were then stained with Alex488- or Alex594-conjugated goat antimouse or rabbit IgG (Invitrogen). After counterstaining with DAPI, sections were examined under a fluorescent microscope (BX53, Olympus, Tokyo, Japan).

### 2.6. Assessment of Oxidative Stress in the Brain

Detection of oxidized protein in the cerebral cortex and hippocampus was performed with the OxyIHC oxidized protein detection kit (MilliporeSigma S7450, Burlington, MA, USA) according to the manufacturer’s instructions. Deparaffinized and rehydrated brain sections were covered with antigen retrieval buffer and incubated in a steamer for 20 min. After incubation with 2,4-dinitrophenylhydrazine (DNPH) solution for 30 min at room temperature, sections were incubated with primary antibody solution, followed by incubation with biotinylated secondary antibody for 30 min at room temperature. After incubation with streptavidin-conjugated HRP, sections were colored with DAB (3,3’-diaminobenzidine)-A/B mixture.

### 2.7. Statistical Analysis

Data were expressed as mean values with standard deviation (mean ± SD). One-way ANOVA was used to evaluate the significance of differences among the groups, followed by a Bonferroni multiple comparison post hoc test. Statistical analysis was performed using Prism statistical software (version 9.2, GraphPad Software, La Jolla, CA, USA). A probability value <0.05 was considered statistically significant.

## 3. Results

### 3.1. Generation of Inducible Neuron-Specific Pnn Depletion Mouse Model

**Figure 1:**
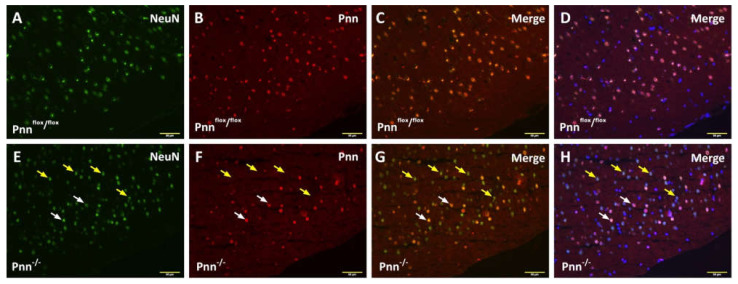
Immunofluorescent staining to examine neuronal Pnn depletion in cerebral cortex. (**A**–**D**) Immunofluorescent staining to detect cerebral Pnn expression in tamoxifen-injected Pnn^flox/flox^ mice (without Cre-ERT2 transgene). (**E**–**H**) Immunofluorescent staining to detect cerebral Pnn expression in tamoxifen-injected CaMKII-CreERT2, Pnn^flox/flox^ mice (Pnn^-/-^). (**A**,**E**) NeuN+ neurons. (**B**,**F**) Pnn+ cells. (**C**,**G**) Merged images. (**D**,**H**) Merged images with DAPI-stained nuclei. In the cerebral cortex of Pnn^flox/flox^ mice, the nuclear distribution of Pnn was found in all NeuN-positive neurons. In Pnn^-/-^ mice, loss of Pnn expression in NeuN+ neurons was observed in cerebral cortex (yellow arrows in **E**–**H**). However, due to the cell type-specific expression of CreERT2, Pnn was also observed in certain cerebral cortical NeuN+ neurons (white arrows in (**E**–**H**)). Scale bar indicates 50 μm.

**Figure 2:**
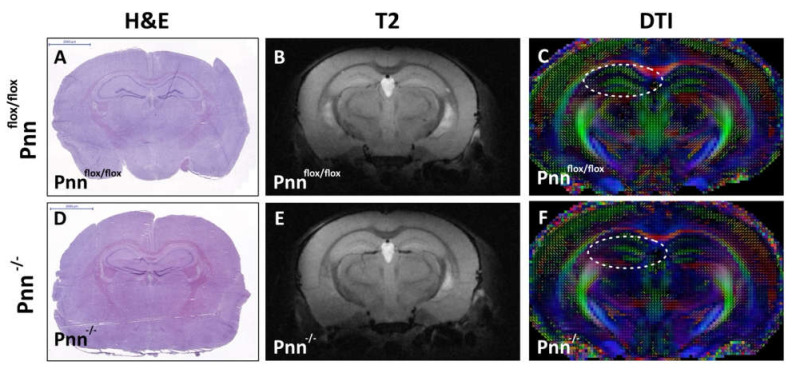
Cerebral histological and MRI examination on mice with neuronal Pnn depletion. (**A**,**D**) Hematoxylin and eosin staining on cerebral cryosections. (**B**,**E**) Magnetic resonance image analysis on 10-week-old male mice with or without neuronal Pnn depletion. (**C**,**F**) Diffusion tensor image to examine white matter tractography in 10-week-old mice with or without neuronal Pnn depletion. Although no significant structural difference was observed, the white matter tractography in hippocampus (white circle in (**C**,**F**)) was altered by neuronal loss of Pnn. f/f, flox/flox mice. -/-, mice with homozygous neuronal Pnn deficiency. Scale bar indicates 2000 μm.

To directly determine the physiological role of Pnn in neurons and the impact of neuronal Pnn depletion in neurological function, we applied an inducible neuron-specific Pnn knock-out mouse model to abolish Pnn expression in neurons. A mouse strain carrying a Pnn conditional allele (Pnn^flox/flox^) was crossed with the mouse strain carrying the Cre-ERT2 transgene driven by the CaMKII promoter (CaMKII-CreERT2, Cre recombinase specifically expressed in excitatory neurons and activated by tamoxifen). Loss of neuronal Pnn was induced by administration of tamoxifen in 6-week-old male mice. Four weeks after tamoxifen injection, immunofluorescent staining was performed to examine the expression and distribution of Pnn in the cerebral cortex. In Pnn^flox/flox^ mice (mice with normal Pnn expression), nuclear distribution of Pnn was observed in all NeuN+ stained neurons (Figure 1A–D). In mice with neuronal Pnn depletion (Pnn^-/-^), loss of Pnn expression was found in most NeuN+ neurons (Figure 1E–H). It is worth noting that, due to the cell type-specific expression patterns of Cre-ERT2, Pnn depletion did not occur in all NeuN+ neurons. To examine the brain structure and white matter tractography in mice with neuronal Pnn depletion, histological examination and magnetic resonance imaging (MRI, T2 and diffusion tensor image) were applied. Although examinations on brain weight (Figure S1) and brain volume (Figure 2. H&E staining and T2 MRI) showed no difference between Pnn^flox/flox^ and Pnn^-/-^ mice, the white mater tractography in the hippocampus was altered by neuronal Pnn depletion (Figure 2F).

### 3.2. Loss of Pnn Regulates the Expression of mRNA Splicing Regulators in Neurons

**Figure 3:**
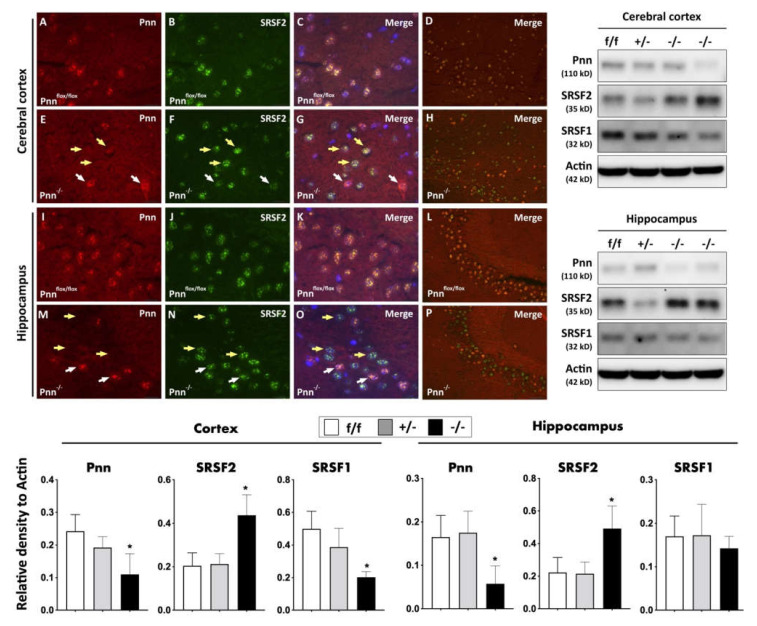
Expression and distribution of splicing regulators in neurons with Pnn depletion. (Upper-left panel) Immunofluorescent staining to examine the expression and distribution of Pnn and splicing regulator SRSF2 in cerebral cortex and hippocampus of mice with or without neuronal Pnn depletion. (Upper-right panel) Western blotting to examine the expression of Pnn, SRSF1, and SRSF2 in cerebral cortex and hippocampus. (Lower panel) Quantitation and comparison of protein expressions of Pnn, SRSF2, and SRSF1 in cerebral cortex and hippocampus. (**A**,**E**,**I**,**M**) Expression of Pnn in cerebral cortex and hippocampus. (**B**,**F**,**J**,**N**) Expression of SRSF2 in cerebral cortex and hippocampus. (**C**,**G**,**K**,**O**) Merged images with DAPI nuclear counter-staining. (**D**,**H**,**L**,**P**) Low-power-field merged images without DAPI staining. Results from immunofluorescent staining further demonstrated the increased expression of SRSF2 in neurons without Pnn expression (yellow arrows), while cells with Pnn expression showed a lower level of SRSF2 expression (white arrows). In Western blotting, results indicated that neuronal Pnn depletion reduced the expression of SRSF1, while the expression level of SRSF2 showed a contrary manner. SRSF1, serine and arginine rich splicing factor 1. SRSF2, serine and arginine rich splicing factor 2. f/f, flox/flox mice. +/-, mice with heterozygous neuronal Pnn deficiency. -/-, mice with homozygous neuronal Pnn deficiency. *n* = 4 for each group. * indicates significance compared with f/f group.

Due to a lack of an RNA recognition motif, it is usually considered that Pnn cannot directly bind to RNA and regulate RNA processing, such as mRNA alternative splicing and nuclear export of mRNA. On the other hand, since Pnn interacts with transcription factors and SR domain-containing RNA binding proteins to regulate gene transcription and mRNA processing, Pnn is suggested to participate in mRNA alternative splicing through regulating activity or expression of splicing regulators. To determine whether Pnn depletion influences the expression of splicing regulators in neurons, we examined the expression levels of two widely expressed splicing regulators with regulatory antagonism, SRSF1 and SRSF2, in the hippocampus and cerebral cortex. Compared to normal mice, a decrease in expression of SRSF1 and an increase in SRSF2 expression were both observed in the cerebral cortex of mice with Pnn depletion (Figure 3). However, the decrease in SRSF1 expression was not observed in the hippocampus with neuronal Pnn deficiency. To further validate the increased expression of SRSF2, we utilized immunofluorescent staining to examine the expression and distribution of SRSF2 in cerebral cortical neurons and hippocampal neurons. Similar to that observed in Western blotting, compared to normal neurons, higher expression of SRSF2 was found in cortical and hippocampal neurons with Pnn deficiency (Figure 3).

### 3.3. Increased Oxidative Stress in Neurons with Pnn Depletion

**Figure 4:**
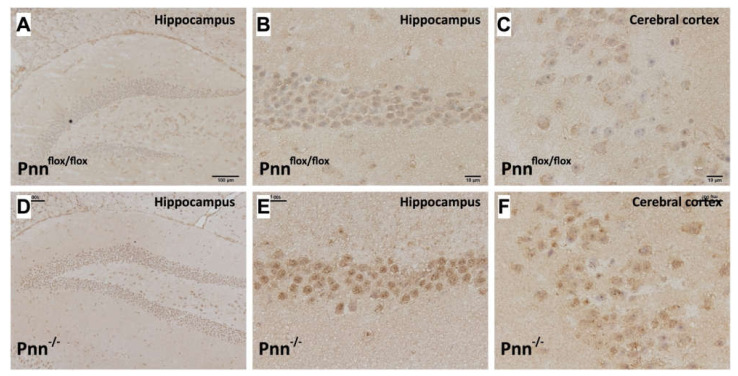
Increased oxidative stress in neurons with Pnn depletion. (**A**–**C**) OxyIHC to detect oxidized proteins in hippocampus and cerebral cortex of Pnn^flox/flox^ mice. (**D**–**F**) OxyIHC to detect oxidized proteins in hippocampus and cerebral cortex of Pnn^-/-^ mice. Results showed that the level of oxidized proteins (brown color) was increased in both hippocampus and cerebral cortex of mice with neuronal Pnn depletion.

**Figure 5:**
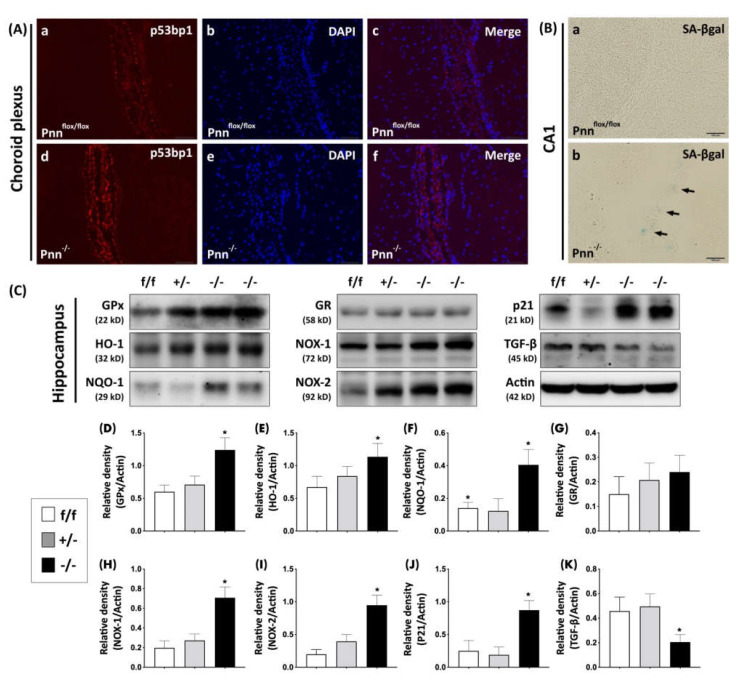
Examination of senescence phenotype in brains with neuronal Pnn depletion. (**A**) Immunostaining to detect p53bp1, a DNA damage marker, in the choroid plexus. (**a**–**c**) Expression of p53bp1 in choroid plexus of Pnn^flox/flox^ mice. (**d**–**f**) Expression of p53bp1 in choroid plexus of Pnn^-/-^ mice. (**B**) Senescence-associated β-galactosidase activity measurement in hippocampus (black arrows) of Pnn^flox/flox^ mice (**a**) and Pnn^-/-^ mice (**b**). (**C**) Western blotting to examine the expression levels of antioxidative proteins (GPx, HO-1, NQO-1), ROS generators (NOX-1, NOX-2), senescence marker p21, and axon initiation protein TGF-β in hippocampus. (**D**–**K**) Quantitation and comparison of protein level of oxidative stress and senescence-associated proteins in hippocampus. Results showed that neuronal Pnn depletion increased DNA damage in choroid plexus, senescence-associated β-galactosidase activity in CA1 of hippocampus, hippocampal oxidative stress as well as p21 expression in hippocampus. p53bp, p53 binding protein 1. GPx, glutathione peroxidase. HO-1, heme oxygenase-1. NQO-1, NAD(P)H quinone oxidoreductase 1. GR, glutathione reductase. NOX-1, NADPH oxidase 1. NOX-2, NADPH oxidase 2. TGF-β, transforming growth factor-β. f/f, flox/flox mice. +/-, mice with heterozygous neuronal Pnn deficiency. -/-, mice with homozygous neuronal Pnn deficiency. *n* = 4 for each group. * indicates significance compared with f/f group.

The regulatory interaction between mRNA alternative splicing and oxidative stress has been considered as a key factor in neuronal injury [16]. To examine whether Pnn not only influences the expression of splicing regulators but also regulates the homeostasis of oxidative stress in neurons, we examined the level of oxidized proteins in brains with OxyIHC. Results showed that higher expressions of oxidized proteins were observed in cortical and hippocampal Pnn-deficient neurons in comparison with normal neurons (Figure 4). To further investigate the regulation of oxidative stress in brains with neuronal Pnn deficiency, we utilized Western blotting to examine the expression level of oxidative stress-associated proteins in the hippocampus. In mice with neuronal Pnn depletion, the cerebral protein expressions of ROS generators, NOX-1 and NOX-2, were increased. Along with the increase in oxidative stress, expression levels of antioxidative proteins, including GPx, HO-1, and NQO-1, were upregulated with Pnn depletion (Figure 5C). However, the expression of another antioxidative protein, GR, was not affected by loss of Pnn in neurons (Figure 5C).

### 3.4. Senescence Phenotype in Brains with Neuronal Pnn Depletion

Both increased oxidative stress and abnormality in RNA processing are considered to be involved in neuronal injury and neurodegenerative diseases [17], which are associated with senescence phenotype in neurons. Therefore, following examinations on cerebral oxidative stress, we examined the senescence-associated β-galactosidase activity in the hippocampus and observed an upregulation of senescence-associated β-galactosidase activity in the hippocampus CA1 of mice with neuronal Pnn deficiency (Figure 5B). Moreover, an intense expression of p53bp1, a DNA damage marker, was also observed in the choroid plexus of Pnn-deficient mice (Figure 5A). In addition, the hippocampal expression of p21, one of the senescence markers, was upregulated in mice with neuronal Pnn deficiency, while the expression of axon initiation protein TGF-β was reduced by Pnn depletion (Figure 5C).

### 3.5. Neuronal Pnn-Depleted Mice Show Exacerbated Brain Infarction

**Figure 6:**
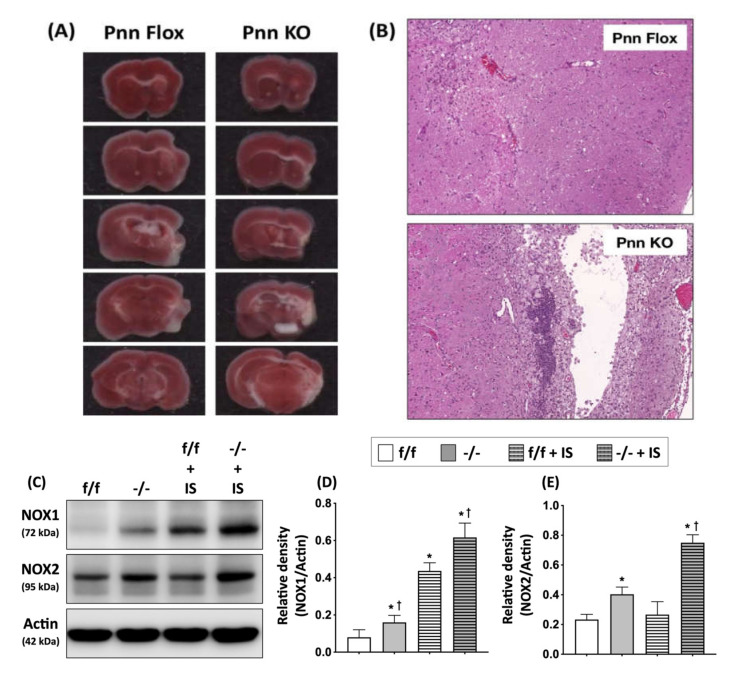
Neuronal Pnn depletion exacerbating brain infarction in mice with cerebral ischemia/reperfusion injury. (**A**) 2,3,5-triphenyltetrazolium chloride (TTC) staining was performed on cerebral slices to identify infarcted region. White color indicates a loss of dehydrogenase activity in the infarcted region. (**B**) To determine the cerebral infarction high-power field, a histopathological examination with H&E staining was performed on cerebral sections. (**C**) Western blotting to ex-amine the expression of NOX-1 and NOX-2 in peri-infarct cortex. (**D**) Comparison of protein levels of NOX-1 in peri-infarct cortex. (**E**) Comparison of protein levels of NOX-2 in peri-infarct cortex. A significant loss of brain tissues, infiltration of immune cells, and increased level of NOXs were observed in mice with neuronal Pnn depletion and ischemic stroke. f/f, flox/flox mice. -/-, mice with homozygous neuronal Pnn deficiency. IS, ischemic stroke. NOX-1, NADPH oxidase 1. NOX-2, NADPH oxidase 1. *n* = 4 for each group. * indicates significance compared with f/f group. † indicates significance compared with f/f + IS group.

To directly examine whether Pnn plays an essential role in protecting neurons against ischemic injury, we applied MCAO-induced cerebral ischemia/reperfusion injury in mice with neuronal Pnn depletion. Histopathological and biochemical examinations were preformed to determine cerebral infarction and activation of apoptotic pathways in brains 21 days post induction of ischemic stroke. In comparison with normal mice, increased infarcted area was observed in mice with neuronal Pnn depletion (Figure 6).

## 4. Discussion

In the present study, we used a mouse model with inducible neuron-specific Pnn depletion to examine the role of Pnn in adult neurons as the stress response to ischemic injury. Unlike Pnn deficiency during embryogenesis, Pnn deficiency does not lead to cell death in neurons (Figure 1). Instead of loss of neurons in the cerebral cortex and hippocampus (Figure 2), Pnn depletion increased oxidative stress in neurons (Figure 4) and triggered senescence phenotype in brains (Figure 5). Moreover, the expression of splicing regulators, SRSF1 and SRSF2, in the hippocampus and cerebral cortex was regulated by neuronal Pnn deficiency (Figure 3). In a mouse model with MCAO-induced cerebral ischemia/reperfusion injury, we also found an increase in infarcted area in mice with neuronal Pnn depletion (Figure 6).

In our previous study, we demonstrated that Pnn regulates the expression level of SRSF1 and SRSF2 in a breast cancer cell line, MCF-7 (Michigan Cancer Foundation-7), and indicated an SRSF1-dependent Pnn deficiency-induced cellular apoptosis [5]. However, whether Pnn regulates expression of splicing regulators in non-dividing and highly differentiated cells, such as adult neurons, remains unknown. In this study, through generation of a neuron-specific Pnn deficiency mouse model, we further proved that the expression level of splicing regulators (e.g., SRSF1 and SRSF2) is directly affected by Pnn depletion (Figure 3). It is noteworthy that Pnn depletion leads to a decrease in SRSF1 and an increase in SRSF2 in neurons. The crosstalk between oxidative stress and RNA binding proteins, particularly those involved in mRNA alternative splicing, is an emerging topic in neuronal injury [17,18,19]. To regulate gene expression of antioxidative proteins through gene transcription and mRNA, alternative splicing is considered as a cellular stress response against oxidative stress [18,20,21]. In an animal model with liver-specific SRSF2 depletion, acute liver failure with elevated oxidative stress was also observed [22], indicating the importance of alternative splicing in cellular redox homeostasis and survival. In this study, we demonstrated an increased level of oxidized proteins in hippocampal and cortical neurons with Pnn deficiency (Figure 4). Upregulated expressions of ROS generator (e.g., NOX-1 and NOX-2) and antioxidant enzymes (GPx, HO-1, and NQO-1) were also observed in the hippocampus with neuronal Pnn depletion. Echoing the finding on expression of splicing regulators in Pnn-deficient neurons, it is reasonable that the increased oxidative stress in Pnn-deficient neurons is mediated by abnormal expression of splicing regulators, such as SRSF1 and SRSF2.

In addition to oxidative stress, the involvement of spicing regulators in the induction or processing of cellular apoptosis was also reported [23,24]. A previous study has indicated that transcription factor E2F1 (E2F transcription factor 1) controls alternative splicing of apoptosis-associated genes through upregulating SRSF2 expression [23]. The inhibition of apoptosis pathways through decreased expression of SRSF2 in renal cancers was also reported [24]. Recent studies further indicated an involvement of SRSF2 in regulating mRNA alternative splicing and expression of SMN, a key protein in inherited spinal muscular atrophy (SMA) [25,26]. On the other hand, SRSF1 shows a protective role against apoptosis through preventing DNA damage and reducing the expression of proapoptotic isoforms of apoptosis-associated proteins [27,28]. Moreover, the antagonist relationship in regulating pre-mRNA alternative splicing between SRSF1 and SRSF2 has also been demonstrated in previous studies [29]. Taken together, the aforementioned reports and findings in the present study all indicate that the exacerbated brain injury post ischemic stroke in Pnn-deficient mice may be mediated by dysregulated expression of splicing regulators, increased oxidative stress, and activating of proapoptotic pathways. However, further studies are needed to clarify the underlying mechanisms.

There are limitations in this study. Recent clinical studies have revealed a critical role of ischemia/reperfusion injury and inflammatory response in the outcome of ischemic stroke [30], while oxidative stress and inflammatory response are considered as a consequence of ischemia/reperfusion injury [31]. However, we did not examine the inflammatory indices in mouse brain with neuronal Pnn depletion and ischemic stroke in the present study. In addition, an RNA-seq to examine the mRNA profiles was not performed to determine whether the expressions of oxidative stress- and apoptosis-associated proteins are regulated by gene transcription and mRNA alternative splicing. Although our previous study has indicated Pnn regulates expression of splicing regulators through a posttranslational regulation, due to the fundamental difference between highly proliferative tumor cells and non-dividing neurons, a cell model study with primary cultured neurons should be applied to clarify the regulatory relationship between Pnn and expression of splicing regulators.

## 5. Conclusions

Through generation of a mouse model with neuron-specific Pnn depletion, we found that Pnn deficiency regulates the expression level of SRSF1 and SRSF2, increases oxidative stress in neurons, and exacerbates ischemia/reperfusion-induced cerebral injury in mice, indicating an important role of Pnn in survival of neurons post ischemic stroke. However, more studies are needed to reveal the underlying mechanism among Pnn depletion, regulation of mRNA alternative splicing, and increased oxidative stress in neurons.

## Data Availability

Data is contained within the article.

## Supplementary Materials

The following supporting information can be downloaded at: www.mdpi.com/article/10.3390/antiox11030466/s1, Figure S1: Gross observation of mice with neuronal Pnn depletion.
