# Supplementary material for: Neuronal Pnn Deficiency Increases Oxidative Stress and Exacerbates Cerebral Ischemia/Reperfusion Injury in Mice
Source: Antioxidants (Basel). 2022 Feb 26;11(3):466. doi: 10.3390/antiox11030466 (PMC8944488; doi:10.3390/antiox11030466)
Supplement: Supplementary file 1 [file antioxidants-11-00466-s001.zip › antioxidants-1572392-supplementary.pdf]

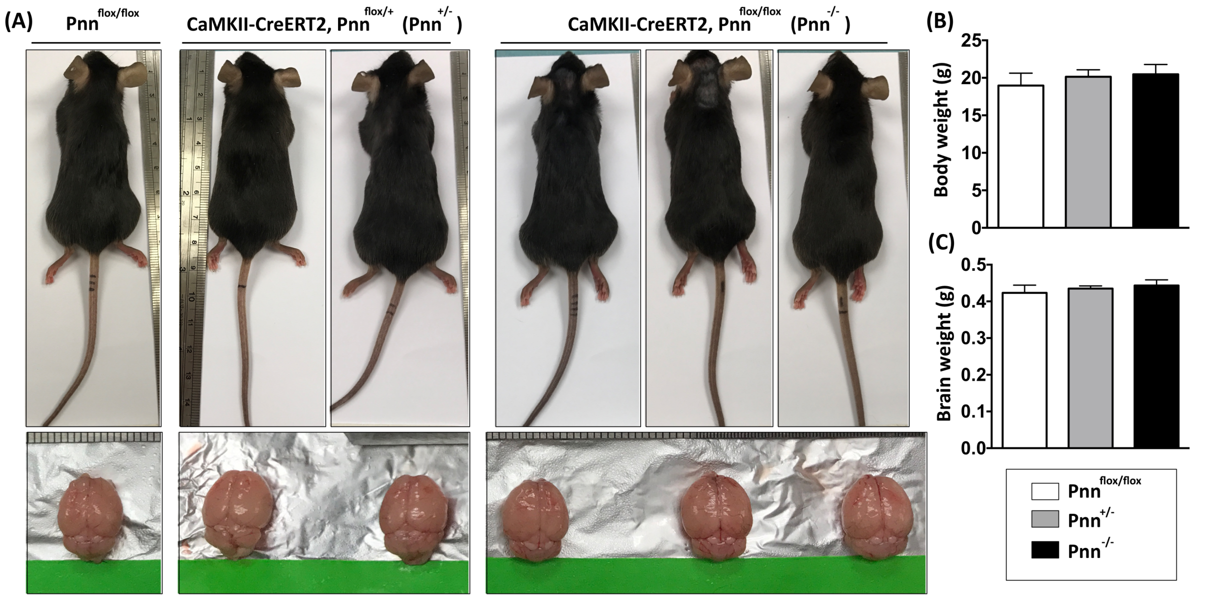

Figure S1. Gross observation of mice with neuronal Pnn depletion

Four weeks after injection of tamoxifen (10-week-old mice), animals were sacrificed for brain tissue collection. The body size (A), body weight (B), and brain weight (C) showed no difference among $Pnn^{flox/flox}$, $Pnn^{+/-}$, and $Pnn^{-/-}$ groups. n=6 for each group.
